# Heterotrophic growth of *Neochloris oleoabundans* using glucose as a carbon source

**DOI:** 10.1186/1754-6834-6-100

**Published:** 2013-07-13

**Authors:** Daniela Morales-Sánchez, Raunel Tinoco-Valencia, John Kyndt, Alfredo Martinez

**Affiliations:** 1 Departamento de Ingeniería Celular y Biocatálisis, Instituto de Biotecnología, Universidad Nacional Autónoma de México, A P 510-3, Cuernavaca, Morelos, 62250, México; 2 College of Science and Technology, Bellevue University, 1000 Galvin Road South, Bellevue, NE, 68005, USA

**Keywords:** *Neochloris oleoabundans*, Heterotrophic growth, Glucose, Glucose transporter, Fed-batch, Nitrate, Lipids, Protein

## Abstract

**Background:**

In comparison with phototrophic growth, heterotrophic conditions can significantly increase growth rates, final cell number and cell mass in microalgae cultures. *Neochloris oleoabundans* is a microalga of biotechnological interest that accumulates lipids under phototrophic and nitrogen-limited conditions. Heterotrophic flask culture experiments were conducted to identify carbon sources that can be metabolized by *N. oleoabundans*, and bioreactor batch and fed-batch (nitrate pulse additions) cultures supplemented with glucose were performed to study the cellular composition of the microalgae under balanced and high C/N ratios (glucose/nitrate).

**Results:**

*N. oleoabundans* was able to grow using glucose and cellobiose as sole carbon sources under strict heterotrophic conditions. Under a balanced C/N ratio of 17 and using bioreactor batch cultures containing 3 g/L glucose, a maximal cell mass of 1.72 g/L was found, with protein being the major cell component (44% w/w). A maximal cell mass of 9.2 g/L was obtained using batch cultures at a C/N ratio of 278. Under these conditions, lipid accumulation was promoted (up to 52% w/w) through N-limitation, resulting in high lipid productivity (528.5 mg/L/day). Fed-batch cultures were performed at a C/N ratio of 278 and with nitrate pulse additions. This condition allowed a maximal cell mass of 14.2 g/L to be achieved and switched the metabolism to carbohydrate synthesis (up to 54% of dry weight), mainly in the form of starch. It was found that transmembrane transport under these conditions was dependent on a proton-motive force, indicating that glucose is transported by a symporter.

**Conclusions:**

*N. oleoabundans* was able to grow under strict heterotrophic culture conditions with glucose or cellobiose as the only carbon source. The glucose used is transported by a symporter system. Batch cultures with a balanced C/N ratio accumulate proteins as the major cellular component; a high C/N ratio significantly increased the dry cell mass and resulted in a high lipid content, and a high cell density was achieved using fed-batch cultures promoting carbohydrate accumulation. These results suggest heterotrophic batch cultures of *N. oleoabundans* as an alternative for the production of proteins or lipids with simple culture strategies and minimal-mineral media supplemented with glucose.

## Background

Recently, several photosynthetic microalgae have been identified as efficient biological systems for producing a wide variety of high-value chemicals and pharmaceuticals, such as phycobiliproteins, astaxanthin and polyunsaturated fatty acids (PUFAs) [1,2]. Consequently, several processes have been developed to obtain some of these compounds on a commercial scale; most of these developments are based on phototrophic growth using CO_2_ as a carbon source [1,3].

Although most microalgae grow photoautotrophically, some are able to grow heterotrophically using organic substrates as sole carbon and energy sources [1]. The heterotrophic growth of microalga depends on the strain and culture conditions, and the consumption of the carbon source depends on the transport or diffusion of the carbon source across the membrane, and the enzymatic processes required for its incorporation into the central carbon metabolism [4]. Compared to photoautotrophic growth, heterotrophic cultivation of microalgae eliminate light requirements, can significantly increase growth rates and cell mass, protein and lipid productivities [5-7]; bioreactor operation and maintenance is relatively simple and can be performed under strict axenic conditions; also cell masses obtained under heterotrophic conditions are higher because the energy density of the carbon source is higher in comparison with carbon dioxide [7] and cell densities can be increased using some culture strategies like fed-batch cultures, leading to a decrease in the costs of biomass harvesting [8,9]. However, heterotrophic cultures also have drawbacks: there is a limited number of microalgal species that can grow heterotrophically; energy expenses and costs by adding an organic substrate are higher; growth can be inhibited by an excess of organic substrate; and light-induced metabolites cannot be produced [7,8]. One of the most notable advantages of the phototrophic cultivation is that under such condition microalgae fixes carbon dioxide and produces oxygen, contributing to the reduction of carbon emissions to the atmosphere [10]; while heterotrophic cultures use an organic carbon source, consumes oxygen and generates some CO_2_ during this kind of cultivations. Furthermore, phototrophic cultures permit the use of non-potable water and not arable land and do not displace food crops cultures [10]. Microalga can grow heterotrophically using the same media components used in phototrophic cultures, but with an organic carbon source instead of using a continuous flow of carbon dioxide and light. However, the cost of the organic carbon source -that is often high- and the production of CO_2_ are major commercial and environmental concerns of heterotrophic cultures. The price of the glucose (obtained from starch that is produced from plants that are cultivated under phototrophic conditions, *e.g.* corn) is in the order of 0.6 US dollars per kg, while the use of carbon dioxide from flue gases can generate some bonus due to the reduction of emissions to the atmosphere [10]; although, additional cleanup steps of the flue gas are likely to be required.

A microalga suitable for heterotrophic culture should have the following physiological abilities: divide and metabolize without light, grow on easily sterilized culture media, adapt rapidly to environmental changes and withstand the hydrodynamic stresses generated in stirred tank bioreactors and peripheral equipment [1,4,8]. Several strains of algae, including *Chlorella protothecoides, Galdieria sulphuraria, Nitzschia laevis* and *Crypthecodinium cohnii* have been studied under heterotrophic growth conditions to achieve high quantities of dry cellular weight (DCW) and fatty acids, or high productivity of valuable chemicals [5,11-13].

The present study was carried out to investigate whether *Neochloris oleoabundans*, which is classified as an oleaginous microalga when cultured under phototropic conditions, is able to grow under heterotrophic conditions using various carbon sources. The following carbon sources were tested to determine whether *N. oleoabundans* can consume them under heterotrophic conditions: glucose, a sugar utilized by most microorganisms and microalgae [7]; cellobiose, a disaccharide consisting of β(1 → 4) linked D-glucose units, which is obtained from cellulose, an agroindustrial byproduct; xylose and arabinose, which are monosaccharides containing five carbon atoms, obtained from the hydrolysis of hemicellulose; sucrose, a disaccharide comprised of glucose and fructose, which is commonly obtained from sugar cane; fructose (fruit sugar), a simple monosaccharide found in many plants and the most water-soluble of all the sugars; lactose, a disaccharide consisting of galactose and glucose, which is found mostly in milk and dairy wastes and has previously been reported as a carbon source for *N. oleoabundans* under mixotrophic conditions [14]; glycerol, which is widely used for the cultivation of microalgae under heterotrophic conditions [7] (currently, glycerol has become very inexpensive because it is an abundant residual byproduct from the biodiesel industry [15,16]); and acetic acid, which is a common carbon source for many microbial species, including microalgae [7,17].

**Figure 1:**
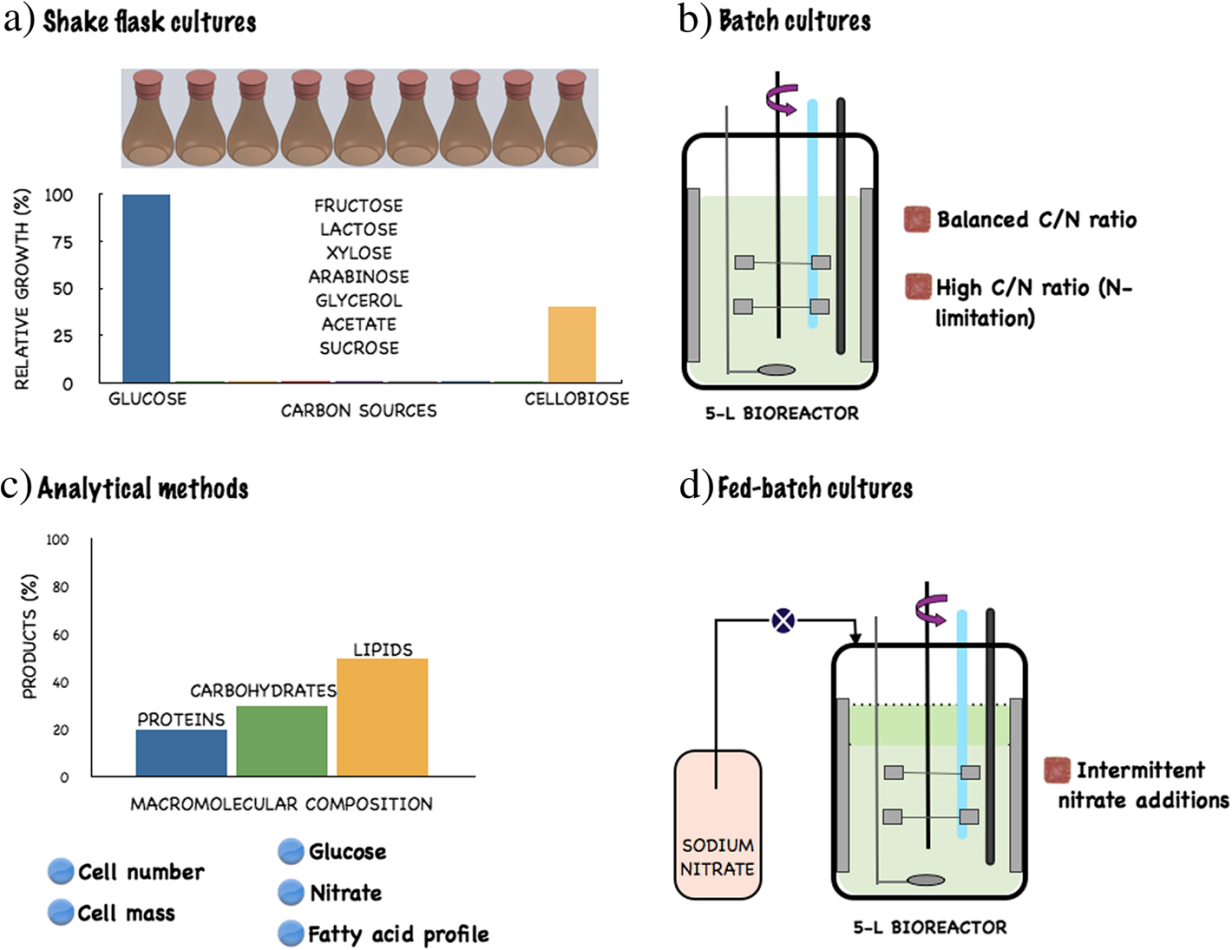
**Schematic representation of the experimental strategy. (a)** Cultures in shake flasks were used to determine the carbon sources that can be metabolized by *N. oleoabundans* under strict heterotrophic conditions and were also used, with glucose, to generate the inocula (Relative growth obtained after 5 days of cultivation). **(b)** Next, glucose was used for batch cultivations in 5-L bioreactors, two different C/N ratios were studied, a balanced ratio of 17 and a high ratio of 278 (nitrogen limited) **(c)** For the batch and fed-batch cultures the following parameters were determined: the cell number and cell mass, the glucose and nitrate consumption, the macromolecular composition of the microalgae (total protein, carbohydrates and lipids) and the fatty acid profile. **(d)** Fed-batch cultures, with intermittent nitrate additions, were performed in 5-L bioreactors.

We characterized the performance of this microalga in batch and fed-batch heterotrophic cultures using glucose as the sole carbon and energy source (Figure 1). In addition, the type of the transmembrane transporter used by *N. oleoabundans* to transport glucose was determined.

## Results and discussion

### Determination of carbon sources metabolized under heterotrophic conditions by *N. oleoabundans*

Results from shake flask cultures indicate that *N. oleoabundans* does not use xylose, arabinose, sucrose, fructose, lactose, glycerol or acetate as carbon sources under strict heterotrophic growth, *i.e.*, dark conditions and mineral media; however, this organism is able to metabolize glucose and cellobiose to grow heterotrophically. Final dry cell weights of 2.3 g/L (dry cell mass productivity of 0.47 g/L/d) and 1.56 g/L (dry cell mass productivity of 0.3 g/L/d) were obtained at 5 days of cultivation of *N. oleoabundans* with 10 g/L of glucose or cellobiose, respectively (relative cell masses obtained in these cultures are reported in Figure 1a). The increase in dry cell weight or cell number and the consumption of xylose, arabinose, sucrose, fructose, lactose, glycerol or acetate was null in a period of 5 days; these experiments were repeated four times obtaining the same results. Hence it was concluded that under strict heterotrophic conditions and using mineral media such carbon sources cannot be metabolized by *N. oleoabundans*.

In a study done by Wu et al. [14] on the production of an extracellular biopolymer by *N. oleoabundans* using glucose or lactose (5 g/L) as a carbon source and a mineral medium, and the same components as the Bold’s Basal Medium (BBM) used in our work, it was demonstrated that the microalga was able to grow on glucose; however, a three-fold lower biomass yield was obtained than that obtained in the present work. Unlike our results, Wu et al. [14] reported that *N. oleoabundans* is able to grow under mixotrophic conditions using lactose as a carbon source. To date, we do not have an explanation for this discrepancy, although we used strict heterotrophic conditions.

Glucose is the preferred carbon source for most living cells on earth. Additionally, glucose has been reported as a carbon and energy source in many heterotrophic cultures of microalgae [5,7,18,19]. Apart from glucose and cellobiose, *N. oleoabundans* apparently does not have the cellular metabolism to transport or catabolize the carbon sources tested. It has been shown with other microalgae that glucose allows higher rates of growth and respiration than do other carbon sources, such as other sugars, sugar alcohols, sugar phosphates, organic acids, and monohydric alcohols [7,20]. Furthermore, in *Chlorella pyrenoidosa*, it was demonstrated that it is possible to generate more ATP under heterotrophic conditions using glucose as an energy source than under phototrophic conditions with light as an energy source [21].

Glucose promotes physiological changes in some microalgae species; for example, in *Chlorella vulgaris*, glucose strongly affects metabolic carbon assimilation pathways, the size of the cells, the quantity of storage materials, such as starch, lipids and protein, and the cellular contents of chlorophyll, RNA, and vitamins [7].

The pathways reported for glucose catabolism in algae are the Embden-Meyerhof and the Pentose Phosphate pathways [15]. The fact that *N. oleoabundans* can grow under dark conditions is noteworthy because some species of microalgae, such as *Dunaliella tertiolecta* and *Prymnesium parvum*, are unable to assimilate glucose in the dark despite the fact that they have the enzymatic activities needed to metabolize this sugar [15].

The observation that *N. oleoabundans* does not grow on the carbon sources tested in this work other than glucose and cellobiose might be explained by: (1) a lack or low activity of certain enzymes, (2) the inability to simultaneously oxidize these compounds and supply the cell with reducing power for biosynthesis, (3) the lack of appropriate permeases, including those located in the plasmalemma and mitochondrial membrane [20], (4) and the putative presence of cellobiohydrolases in the enzymatic repertory of *N. oleoabundans*. Because the microalga *N. oleoabundans* is not adapted to elevated osmolarity, unlike marine microalgae that live in seawater and saline ponds [7,15], it can be speculated that *N. oleoabundans* could not grow using other carbon sources, such as glycerol.

Due to the higher cell density concentration obtained with glucose compared to cellobiose, we decided to characterize the growth, nutrient assimilation and macromolecular cell composition of *N. oleoabundans* under more controlled environmental conditions using batch cultures in 5-L bioreactors with glucose as a carbon and energy source (Figure 1).

### Batch cultures with an equilibrated C/N ratio allows protein accumulation

**Figure 2:**
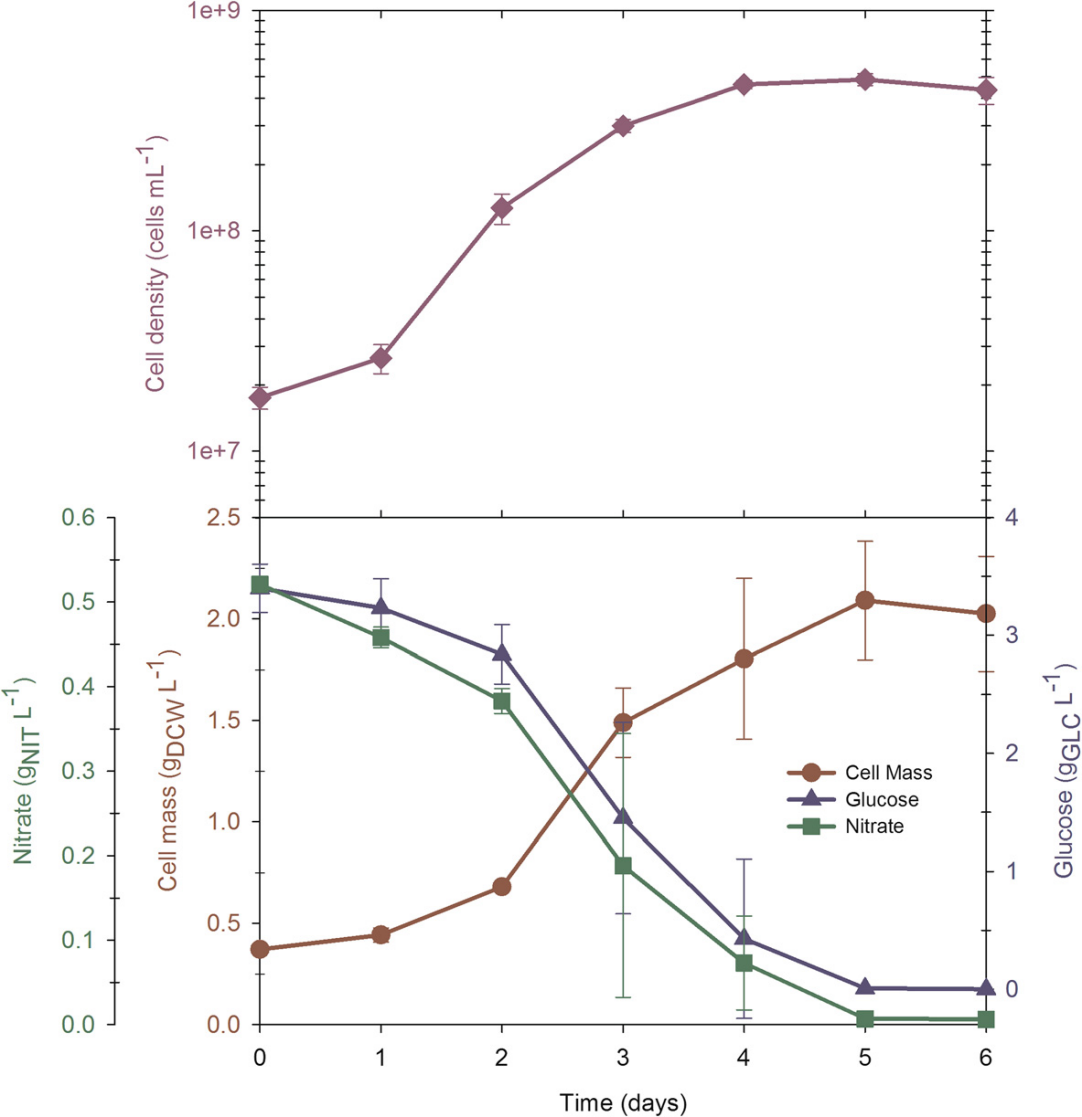
**Batch cultivation of *N. oleoabundans *with a balanced C/N ratio (C/N = 17).** The experiments were performed in triplicate and the results in the figure show the average and standard deviation.

A carbon and nitrogen balance calculation was performed to determine the number of moles of carbon and nitrogen required for the growth of *N. oleoabundans* without limiting one of these nutrients when cultured with BBM salts. Batch cultures were developed with 3 and 0.5 g/L of glucose and sodium nitrate, respectively, to equilibrate the atomic ratio of carbon and nitrogen at 17 (*i.e.*, a C/N ratio of 17); such value was estimated based in the elemental composition of *N. oleoabundans* cell reported by Pruvost et al. 2009 [22] and previous range values reported in the literature for other microalgal species [9,19,23]. To avoid any nutrient limitation other than those of glucose or sodium nitrate, all components of the BBM media were added at twice the normal concentration. As shown in Figure 2, a cell mass concentration of 1.72 g/L was attained, representing an increase by one order of magnitude compared to that present at the beginning of the culture period. The specific growth rate (μ), which was calculated based on the exponential part of the growth phase, was 0.05/h (Table 1); this value is equivalent to a duplication time (t_D_) of 13 h. Exhaustion of the growth-limiting nutrients (glucose and nitrate) occurred by 5 days of culture, ending exponential growth.

**Table 1:** Kinetic and stoichiometric parameters of the cultures evaluated in this work

**Culture**	**μ (day**^**-1**^**)**	**X**_**MAX **_**(g**_**DCW **_**L**^**-1**^**)**	**Y**_**X/GLC **_**(g**_**DCW**_**g**_**GLC**_^**-1**^**)**	**Y**_**X/NIT **_**(g**_**DCW**_**g**_**NIT**_^**-1**^**)**	**Q**_**X **_**(mg**_**DCW**_**L**^**-1**^ **day**^**-1**^**)**	**Q**_**PROT **_**(mg**_**PROT**_**L**^**-1**^ **day**^**-1**^**)**	**Q**_**LIP **_**(mg**_**LIP**_**L**^**-1**^ **day**^**-1**^**)**	**Q**_**CARB **_**(mg**_**CARB**_**L**^**-1**^ **day**^**-1**^**)**
**Batch C/N = 17**	0.05 ± 0.001	1.72 ± 0.30	0.57 ± 0.10	3.3 ± 0.56	344.0 ± 8.0	150.3 ± 1.7	82.6 ± 4.8	106.3 ± 1.0
**Batch C/N = 278**	0.05 ± 0.004	9.2 ± 0.15	0.62 ± 0.08	4.8 ± 0.43	1,022.2 ± 44.4	147.2 ± 5.1	528.5 ± 7.2	340.9 ± 4.6
**Fed-batch**	0.02 ± 0.002	14.2 ± 0.46	0.3 ± 0.14	3.1 ± 0.67	1,420.0 ± 50.0	164.7 ± 4.26	478.5 ± 7.1	770.1 ± 6.7

μ: Specific growth rate.

X_MAX_: Maximum cell mass produced.

Y_X/GLC_: Yield of biomass on glucose when the culture was in nutrient sufficient and during the feed in the case of fed-batch culture.

Y_X/NIT_: Yield of biomass on nitrate when the culture was in nutrient sufficient and during the feed in the case of fed-batch culture.

Q_X_: Biomass productivity at maximum total cell mass produced.

Q_PROT_, Q_LIP_, Q_CARB_: Protein, lipid or carbohydrate productivity at maximum cell mass produced.

Compared with a phototrophic culture of *N. oleoabundans* grown in the same culture media [22], heterotrophic cultivation decreased the duplication time by half and increased the cell density 3.5 times.

Throughout the cultivation time, the ratios between lipids, proteins and carbohydrates remained constant, with most of the weight representing protein (on average 42.5% (w/w), Table 2). Generally, the C/N ratio may influence cell composition by controlling the switch between protein, lipid and carbohydrate syntheses [9]. Due to this equilibrated C/N ratio, resulting from the carbon and nitrogen balance, the cell was not under any type of nutrient stress (until the fifth day) that could promote the accumulation of reserve metabolites; hence, the main macromolecule found in these cultures was protein, with an overall volumetric productivity of 150.3 mg/L/day (Table 1).

**Table 2:** **Macromolecular composition of *N. oleoabundans *cells from batch and fed-batch cultures**

**Culture**	**Proteins (g**_**PROT **_**g**_**DCW**_^-1^*100)		**Carbohydrates (g**_**CARB **_**g**_**DCW**_^-1^*100)		**Lipids (g**_**LIP **_**g**_**DCW**_^-1^*100)	
	**S**	**M**	**S**	**M**	**S**	**M**
**Batch C/N = 17**	41.2 ± 0.4	43.7 ± 1.9	31.7 ± 1.3	30.9 ± 3.5	24.8 ± 0.3	24.0 ± 0.9
**Batch C/N = 278**	42.9 ± 1.7	14.4 ± 1.2	32.1 ± 0.4	33.3 ± 0.5	23.3 ± 1.2	51.7 ± 1.7
**Fed-batch**	40.7 ± 1.4	11.6 ± 0.9	27.5 ± 2.0	54.2 ± 0.1	27.5 ± 1.9	33.7 ± 0.6

S: at the start of the culture.

M: at the time of maximum cell mass produced.

**Figure 3:**
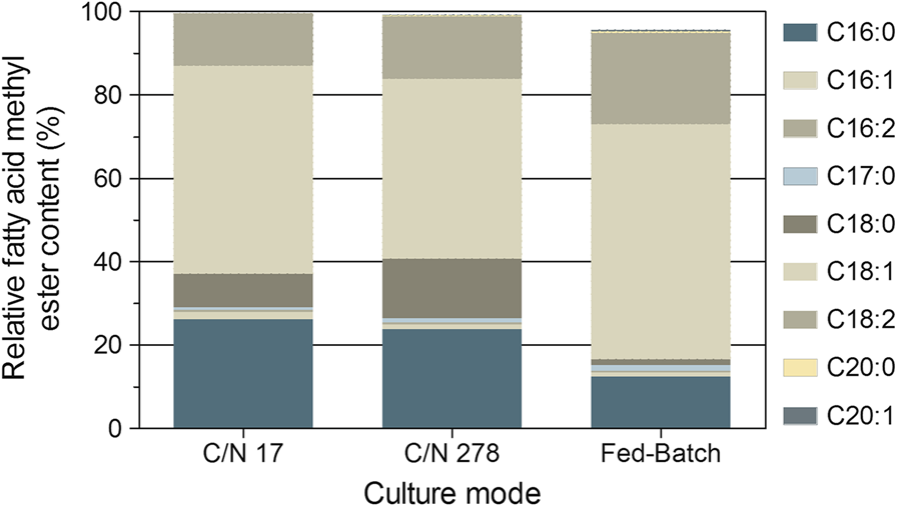
**Fatty acid methyl ester profile in batch cultures with a C/N = 17 and C/N = 278 and fed-batch culture with an initial C/N = 278.** The determinations were performed in triplicate and the results in the figure show the average.

The lipid content was 23% at the sixth day of the culture, with octadecenoic acid (oleic acid, C18:1) as the main component (49.9%; Figure 3) of fatty acids, followed by hexadecanoic acid (palmitic acid, C16:0, 26.1%), octadecadienoic acid (linoleic acid, C18:2, 12.7%) and octadecanoic acid (stearic acid, C18:0, 8.0%). A similar fatty acid composition has been reported by Gouveia et al. [24] for *N. oleoabundans* grown under phototrophic conditions. Cerón et al. [25] stated that these types of lipids act mainly as storage in microalgae.

### Batch cultures with high C/N ratios promotes lipid accumulation

**Figure 4:**
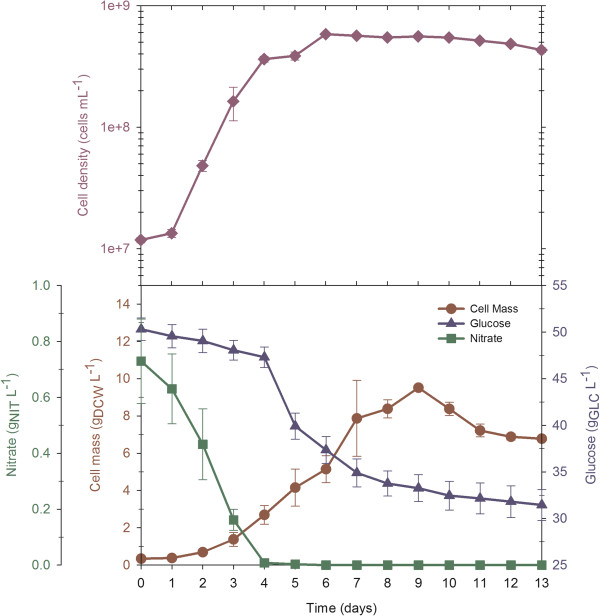
**Batch cultivation of *N. oleoabundans *with a high C/N ratio (C/N = 278).** The experiments were performed in duplicate and the results in the figure show the average and standard error.

To investigate the effect of high glucose concentration (50 g/L) and nitrogen limitation on growth and cell composition, batch cultures with a C/N ratio of 278 were analyzed. To avoid any nutrient limitation other than that of nitrate, all components of BBM were added at twice the normal concentration. The results presented in Figure 4 show that *N. oleoabundans* can grow at high glucose concentration (50 g/L) with a DCW yield of 0.62 g/g (Table 1). The growth rate obtained under these conditions was the same as that using 3 g/L glucose (0.05/h, see Table 1). When these cultures entered into N-limitation (after day 4), the cell density did not increase; but there was a 4-fold increase in the DCW (Figure 4).

During the first four days of cultivation, *i.e.*, during the ‘no nitrogen limitation’ phase where cell density exponentially increased, the ratio of lipids, proteins and carbohydrates was constant, being quite similar to the values for the batch culture with 3 g/L glucose. At day 9, *i.e.*, during nitrogen limitation, lipid accumulation was promoted, up to 50% of the DCW (Table 2). This increase in lipid content is indicative of a change in metabolism toward the synthesis of carbon and energy into reserve metabolites. Some green algae and diatoms can store carbon and energy in the form of lipids (*i.e.,* triacylglycerides, TAGs) under certain stress-inducing culture conditions [26,27], and this behavior has been extensively reported in *N. oleoabundans* when cultivated in phototrophic conditions under N-limitation [22,24,28,29]; however, to the best of our knowledge, this is the first time that such study has been performed for *N. oleoabundans* under nitrogen-limited heterotrophic conditions.

Previous reports have shown that a high C/N ratio promotes lipid accumulation for several other microalgae under heterotrophic conditions [9]. Mainul et al. [23] found that when the C/N ratio in heterotrophic cultures of *Cryptococcus curvatus* was increased from 25 to 70, the oil content increased from 18% to 46%. At a high C/N ratio, the synthesis of nitrogen-containing compounds such as protein and nucleic acids is reduced, and cells enter into the stationary phase and begin to accumulate storage lipids [19]. Unlike other reserve metabolites such as carbohydrates, lipids are preferred as reserve metabolites under prolonged nutritional stress conditions. This has been ascribed to their higher reduction state, hydrophobic character and the fact that they can be efficiently packed into the cell and generate high amounts of energy upon oxidation, thus constituting the best reserve for rebuilding the cell after the nutritional stress has been alleviated [30-32]. The reported values for global lipid productivities in heterotrophic cultures range from 54 mg/L/day for *Chlorella vulgaris* to as high as 600 mg/L/day for *Schizochytrium limacinum* SR21 [33]. Our results indicate a global lipid productivity of 528.5 mg/L/day for nitrogen-limited cultures. This value is consistent with the values presented above for other microalgae and is approximately 4.9 times higher than that achieved in phototrophic cultures of *N. oleoabundans*[28].

As seen in Figure 3, the fatty acid profile of *N. oleoabundans* at a C/N ratio of 278 is very similar to that obtained at a C/N ratio of 17, but the relative content of stearic acid (C18:0) is higher and the relative content of oleic acid (C18:1) is lower for cells cultured at a C/N of 278, but C18:1 still is the principal fatty acid component. Furthermore, C20 fatty acids (arachidic acid, C20:0 and gadoleic acid, C20:1) constituted less than 1% (of the fatty acid content) under nitrogen limiting conditions.

### Fed-batch cultures with nitrate pulse additions switches metabolism to carbohydrate accumulation

**Figure 5:**
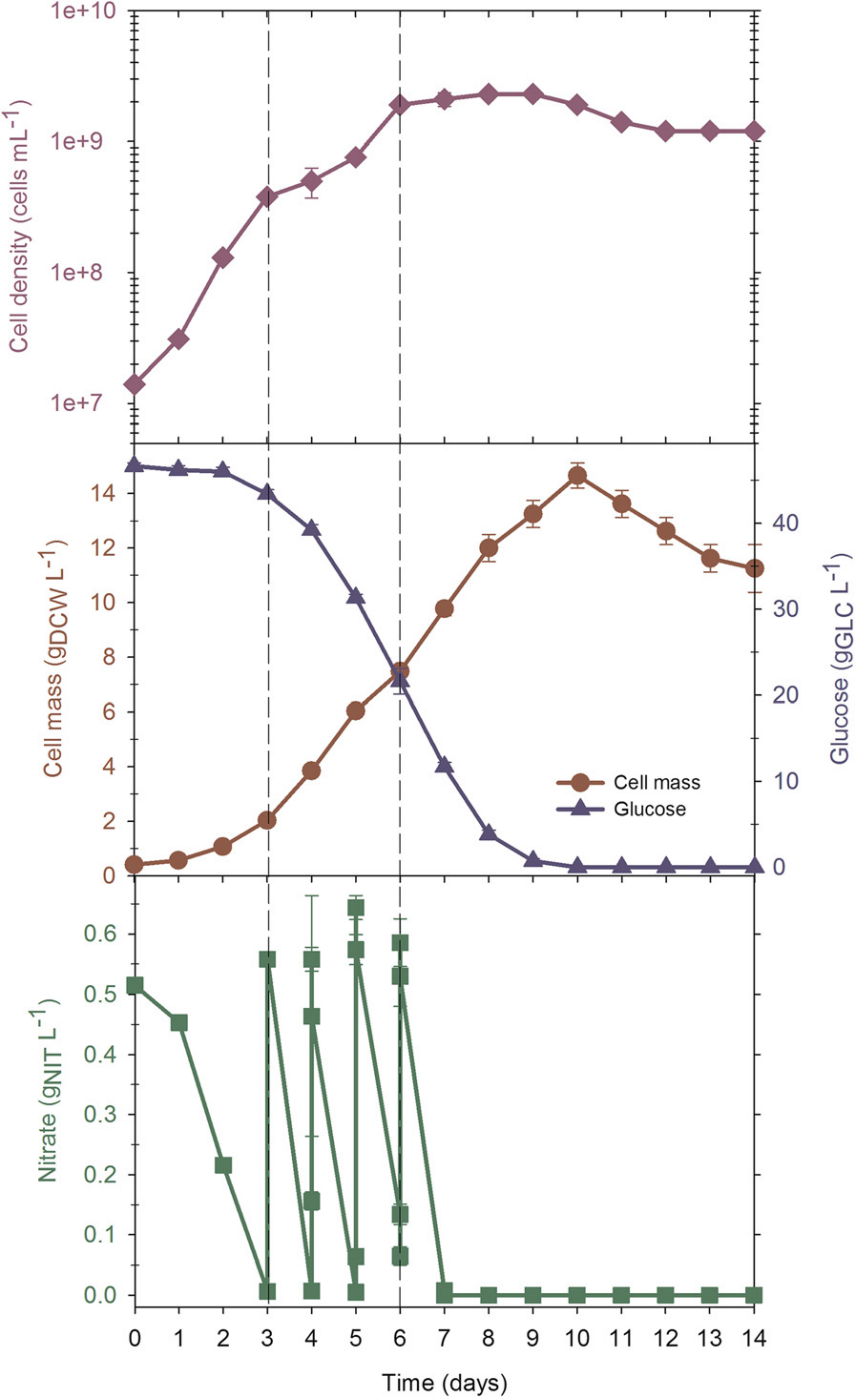
**Fed-batch cultivation of *N. oleoabundans *with an initial C/N = 278 and nitrate pulse additions.** Vertical dashed lines indicate the start and end of the feeding stage. The experiments were performed in duplicate and the results in the figure show the average and standard error.

Fed-batch cultures with ‘nitrate pulse additions’ were performed to obtain a high cell density culture and determine the macromolecular composition of *N. oleoabundans* under such conditions*.* As described in the Methods section, this culture started with a working volume of 3.5 L (50 g/L glucose) in the bioreactor and consisted of three stages (Figure 5). First, a batch culture stage of three days was developed. When the sodium nitrate concentration decreased to a level of 0.05 g/L, the feed stage was initiated; the nitrate feed was manually controlled to maintain a residual nitrate concentration between 0.10 and 0.6 g/L. This profile was developed to maintain a specific rate of nitrate consumption during the fed-batch culture, close to its maximum value. To prevent nutrient limitation other than nitrate, all BBM medium salts (at half of the usual concentration) were also added each time nitrate was supplemented. When a DCW concentration of 14.2 g/L was reached, the final stage was initiated by ending the supply of nitrate (and BBM salts) with the purpose of developing a stationary, nitrogen-limited phase. The last nitrate addition was performed on the sixth day of cultivation, when 21.6 g/L of glucose remained in the medium; nitrate was depleted on the seventh day, and the residual glucose concentration was 11.7 g/L at this time. The final stage was completed when the residual glucose was depleted.

A maximum biomass concentration of 14.2 g/L dry weight was reached at 10 days of cultivation, when glucose was depleted (Figure 5). During the batch and fed-batch stages, cell density increased exponentially, and the estimated specific growth rates were 0.05 and 0.02/h, respectively (Table 1). When nitrate was depleted, cell density no longer increased, but the DCW continued to increase (Figure 5); this finding might suggest the formation and accumulation of a reserve metabolite from metabolism of the consumed glucose. As expected, this culture reached a higher cell mass, approximately eight and two times higher than batch cultures with C/N ratios of 17 (3 g/L glucose) and 278 (50 g/L glucose), respectively.

Table 2 shows the cellular composition of *N. oleoabundans* in the fed-batch culture. While the cells were under nutrient-sufficient conditions, the major cellular fraction was protein. During the third stage (at 7 days), while the cells were under nitrogen limitation but still had sufficient glucose, the cells accumulated carbohydrates with an overall volumetric productivity of 770.1 mg/L/day (Table 1). The carbohydrates were hydrolyzed with α-amylase and gluco-amylase, and the following monosaccharides were found: glucose (with a relative content of 90%, w/w), xylose (6% w/w) and arabinose (~1% w/w). Because the carbohydrates were hydrolyzed with enzymes that digest starch, it is suggested that the main content of these carbohydrates is starch. These results indicate that stages of low and relatively high levels of nitrate over short periods promoted carbohydrate rather than lipid accumulation; carbohydrates are most likely preferred as reserve metabolites under short-term nutritional stress conditions, similar to the situation in plants. In *Arabidopsis*, as in most vascular plants, starch plays an important role in the day-to-day carbohydrate metabolism of the leaf. Glucose produced during photosynthesis is stored mainly in the form of starch granules in the chloroplast, this storage material is used to change the availability of photosynthates obtained during the diurnal cycle of light and dark. Starch that has accumulated during the day through light reactions is degraded during the subsequent night through Calvin cycle or dark reactions, providing a constant supply of carbohydrate in the absence of photosynthesis. Therefore, the plant starch can be seen as a short-term carbohydrate reservoir and its frequency termed transitory starch [34].

As seen in Figure 3, the fatty acid profile of *N. oleoabundans* in fed-batch cultures was different than that in the batch cultures. The saturated fatty acid content was reduced (palmitic acid, C16:0 and stearic acid, C18:0), but longer-chain and unsaturated fatty acids (oleic acid, C18:1 and linoleic acid, C18:2) were synthesized in higher proportions; oleic acid (C18:1) was again the principal fatty acid component. In addition, C:20 fatty acids (arachidic acid, C20:0 and gadoleic acid, C20:1) again represented less than 1% of total fatty acids in the fed-batch culture.

### Transmembrane glucose transport and chlorophyll content

**Figure 6:**
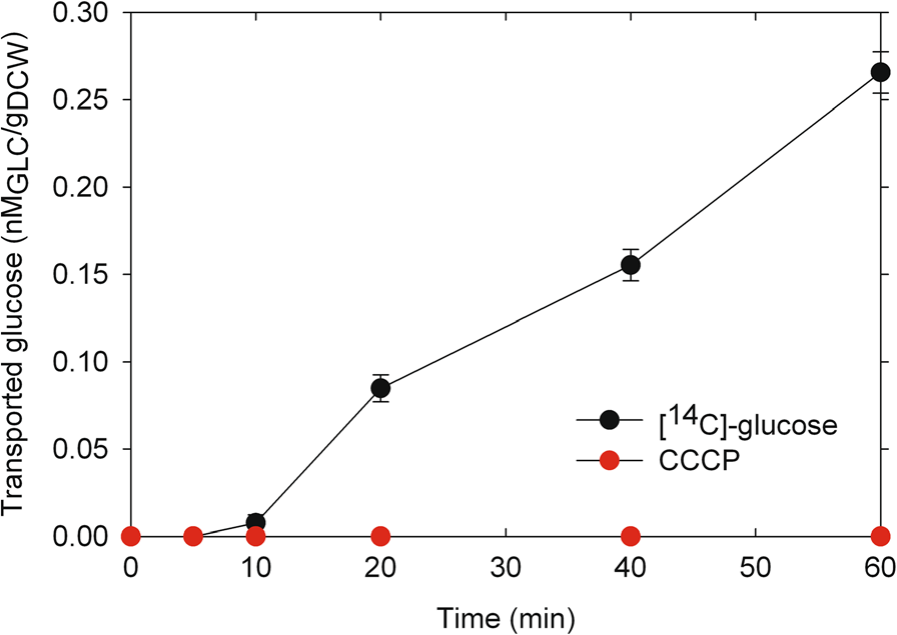
**Transport kinetic of [**^**14**^**C]-glucose with the ionophore carbonyl cyanide m-chlorophenyl hydrazone (red circle) and with [**^**14**^**C]-glucose alone (black circle).** The determinations were performed in triplicate and the results in the figure show the average and standard deviation.

Transmembrane glucose transport was investigated in cells growing in batch cultures containing 3 g/L glucose and [^14^C]-glucose tracer; the ionophore carbonyl cyanide m-chlorophenyl hydrazone (CCCP) was incorporated as a proton gradient decoupler. It is known that CCCP causes an uncoupling of the proton gradient, which is established during the normal activity of electron transport in the cell wall; if a carbon source is transported through a symporter system then CCCP will uncouple its entrance into the cell. As shown in Figure 6, when added to *N. oleoabundans* cells CCCP inhibits the glucose transport across the membrane, indicating that the cells can transport glucose only in the presence of a proton gradient; hence suggesting that this microalga uses a glucose symporter to transport this sugar into the cell. Calculations based on the rates of consumption and uptake suggest that glucose consumption depends on the transport capacity of the cell because the glucose consumption and transport rates are very similar (8 μmol/g/min and 5 μmol/g/min, respectively).

As in *Chlorella* cells, transmembrane transport in *N. oleoabundans* is proton-motive force-dependent, indicating that the transporter system is a glucose symporter. It has been demonstrated that *Chlorella* cells possess an inducible active hexose/H^+^ symport system responsible for the uptake of glucose from the medium [13,35-37]. This system transports sugars and protons with 1:1 stoichiometry, and the cell consumes one molecule of ATP per molecule of sugar transported [13,37]. With glucose as the inducer, the minimum time necessary to induce the synthesis of the hexose/H ^+^ symport system proteins in *Chorella* is 15–18 min [13,37,38].

The total chlorophyll content in cells was determined at the fifth day of batch cultures using the spectrometric method reported by Pruvost et al. [22]. Although cultures were performed under strict dark conditions, the total chlorophyll content ranged from 1.0–1.5% (μg chlorophyll/μg DCW). Such values are 2.3–3.5-fold lower than values found under phototropic conditions [22]. However, the quantities of chlorophyll found under heterotrophic conditions indicate a constitutive expression of the photosynthetic system.

## Conclusions

*N. oleoabundans* was able to grow under strict heterotrophic cultures conditions with glucose or cellobiose as the only carbon source; however, no growth was obtained using xylose, arabinose, fructose, sucrose, lactose, glycerol or acetic acid. Transport studies indicate that glucose is transported by a symporter system, although further study is needed to determine the identity of this transporter. Using different cultivation strategies with variable C/N ratios under controlled conditions (pH, temperature and aeration rate) in a 5-L bioreactor allows metabolism switching. Batch cultures with balanced C/N ratios (17) accumulate proteins (up to 44% w/w) as the major cell component; a high C/N ratio (nitrogen limitation during the stationary phase) significantly increases the dry cell mass and yields a high lipid content (up to 52% w/w). High cell density and carbohydrate accumulation (up to 54% (w/w), mainly starch) were achieved using fed-batch cultures (50 g/L initial glucose and the addition of nitrate pulses). These results suggest heterotrophic batch cultures of *N. oleoabundans* as an alternative for the production of proteins or lipids with simple culture strategies and mineral media supplemented with glucose, which could potentially be obtained by hydrolyzing the glucan fraction of lignocellulose.

## Methods

### Algal strain, media and inocula development

The microalgae *Neochloris oleoabundans* UTEX 1185 was obtained from the Algae Culture Collection of the University of Texas (Austin, TX, USA). Stock cultures were maintained by sub-cultivation in 250-mL shake flasks containing 100 mL of mineral medium (Bold’s Basal Medium; BBM) supplemented with 10 g/L glucose in the dark. Cultures were incubated in an orbital shaker (300 rpm, 25°C; Barnstead MaxQ 4000, USA). BBM [28] comprises the following salts (in mM): NaNO_3_ 2.94,MgSO_4_ · 7H_2_O 0.3, K_2_HPO_4_ 1.29, KH_2_PO_4_ 0.43, CaCl_2_ 0.17, NaCl 0.43, Na-EDTA 0.171, KOH 0.553, and H_3_BO_3_ 0.185, and the following trace elements (in μM): FeSO_4_ · 7H_2_O 18, ZnSO_4_ · 7H_2_O 30.7, MnCl_2_ · 4H_2_O 7.3, CuSO_4_ · 5H_2_O 6.3, MoO_3_ 4.9 and CoNO_3_ · 6H_2_O 1.7 dissolved in H_2_SO_4_ 10.2 μM. All medium components were heat-sterilized (121°C, 20 min). To prepare the inocula, *N. oleoabundans* was cultivated in 250-mL Erlenmeyer flasks with 100 mL of BBM at an initial pH of 7 and at 25°C and 300 rpm. All cultures in flasks and bioreactor were initiated with a 10% (v/v) inoculum, roughly equivalent to an initial cell number of 1 × 10^7^−2 × 10^7^ cells/mL or 0.4 g_DCW_/L.

### Analysis of carbon sources that support heterotrophic growth of *N. oleoabundans*

Cultures in 250-mL Erlenmeyer flasks were used to test common carbon sources that are used for the heterotrophic growth of microalgae or bacteria*.* The carbon sources studied were glucose, xylose, arabinose, fructose, sucrose, lactose, cellobiose, glycerol and sodium acetate. Culture conditions were similar to those used for inocula development and included 10 g/L of the carbon source (except for sodium acetate, which was used at 5 g/L), and the cultures were incubated for 5 days.

### Cultivation in bioreactors

A 5-L bioreactor (LSL Biolafitte, USA) was used for batch and fed-batch cultivation of heterotrophic *N. oleoabundans*. Initial working volumes for the batch and fed-batch cultures were 4 and 3.5 L, respectively. The culture conditions for both operation modes were as follows: 25°C, pH7.0 (adjusted by the automatic addition of 1 N H_3_PO_4_ and 1 N KOH) and 10% (v/v) of the initial inoculum. To determine if the *N. oleoabundans* cells are sensitive to the stirring and/or aeration rates, a preliminary set of experiments were performed using 1 L of air/L of medium/min and three different stirring speeds: 150, 300 and 600 rpm. The growth of the cells, measured as cell density (cells/mL) and dry cell mass, was the same for the three set of experiments, indicating that no mechanical stress was developed at such conditions. Hence batch cultures were developed at 300 rpm (using three Rushton Turbines with 6 blades), and an aeration rate of 1 L of air/L of medium/min; under these conditions, the dissolved oxygen was always greater than 80% of the air saturation value. An initial sodium nitrate concentration of 0.5 g/L was used for the batch cultures, and two sets of experiments were performed with initial glucose concentrations of 3 and 50 g/L, *i.e.*, equivalent to C/N ratios of 17 and 278, respectively.

Kinetic and stoichiometric parameters (specific growth rate; maximum cell mass produced; yield of biomass on glucose; yield of biomass on nitrate; and volumetric productivities for biomass, proteins, lipids or carbohydrates) of the 17 C/N ratio batch culture were determined at mid-log phase. In the 278 C/N ratio batch cultures, these parameters were calculated during the first four days of culture, *i.e.,* when the cultures were not limited by the carbon and/or nitrogen sources.

The initial medium (3.5 L) for the fed-batch cultures also contained 0.5 g/L sodium nitrate and 50 g/L glucose. The consumption of nitrate was previously determined from batch cultures; the times and quantity of sodium nitrate added were based on these values. The specific rate of consumption of sodium nitrate was determined, and the fed-batch cultures were fed based on this rate with intermittent sodium nitrate additions to maintain the concentration between 10 and 600 mg/L. In these cultures, the initial values of agitation and aeration were 300 rpm (using three Rushton Turbines with 6 blades) and 1 L of air/L of medium/min, but the dissolved oxygen was maintained above 20% air saturation by stepwise increments in the agitation speed and airflow rate. For the fedbatch cultures, a mass balance, considering the volume of liquids added (nitrate solution, acid and base used for pH control, and nutrient additions) and removed (sampling and evaporation) was performed to correct the actual values for the cell density and cell mass. Kinetic and stoichiometric parameters of the fed-batch cultures were determined during the feeding phase (pseudo-stationary feeding state) of the culture.

To detect any interference in glucose consumption samples from all cultures (flasks and bioreactors) were tested every day for the presence of contaminants, spreading several dilutions of the broths into solid-rich-media (1.5% agar, 1% tryptone, 0.5% yeast extract and 1% sodium chloride); cultures were discarded if contaminants were detected in solid media after 1–3 days of incubation at 37°C.

### Analytical methods

The cell density (cells/mL) was determined by direct counting using a Neubauer chamber and a light microscope equipped with a 40× objective. The algal dry cell weight (DCW) was evaluated gravimetrically by filtration through a pre-dried and pre-weighed 0.45-μm pore size nitrocellulose membrane filter (Millipore).

Lipids were extracted using solvents and gravimetrically quantified as reported by Band et al. [39]. Briefly, samples with culture media were centrifuged (14,000 × g, 15 min, 4°C), and the cell pellet was resuspended in methanol/dichloromethane (2:1; v/v) containing 0.5 mg of butylated hydroxytoluene and stored at 4°C for at least 12 h. The cell debris was separated by centrifugation (14,000 × g, 15 min, 4°C). After centrifugation, the lipid-containing supernatant was transferred to another tube, and the residue was extracted twice with 4 mL of methanol/dichloromethane (1:1; v/v). Methanol from the solution containing the extracted lipids was separated by adding deionized water. To remove residual water, the resulting organic phase (containing lipids, dichloromethane and residual amounts of water) was filtered through anhydrous sodium sulfate. Finally, solvents were removed using an evaporator (Waterbath B-480 Büchi, Switzerland) at 40°C and atmospheric pressure, and the lipids were gravimetrically quantified.

Carbohydrate content was determined using the phenol-sulfuric acid method after acid hydrolysis of the sample [40], and the protein content was determined using the Lowry method after alkaline hydrolysis of the sample [41].

Glucose was determined in the culture supernatants using a biochemical analyzer (YSI 2700 Select, Yellow Spring Instruments, Ohio, USA). Nitrate concentration was quantified using an ion-selective probe calibrated with sodium nitrate standards (10, 100, and 1,000 mg/L; Ion Concentration Controller Microprocessor Based IC 7685, B&C Electronics Srl, Lombardy, Italy; ISE: Nitrate NO31502, Van London-Phoenix Co, Texas, USA).

After lipid extraction, the total carbohydrates found in *N. oleoabundans* cells from fed-batch cultures were hydrolyzed using α-amylase (Liquozyme SC DS, Novozymes, Franklinton, NC, USA) and gluco-amylase (Spirizyme fuel, Novozymes, Franklinton, NC, USA); the carbohydrate composition (glucose, xylose and arabinose) was determined by HPLC analysis (Waters U6 K, Millipore Co., Milford, MA, USA) using an Aminex HPX-87H ion exclusion column (300 × 7.8 mm; Bio-Rad Laboratories, Hercules, CA, USA), and a 5 mM H_2_SO_4_ solution was used as the mobile phase (0.5 mL/min) at 60°C. A refractive index detector was used to identify the sugars in the hydrolysate (Model 2410, Waters, Millipore Co).

To determine the fatty acid composition, oil samples were chemically derivatized using the boron trifluoride method [10]. Briefly, 10 mg of a sample of total lipids were processed with 2 mL of NaOH (0.3 N-methanol 90% v/v) at 75°C during 2 hours. The non saponifiable lipids (upper layer) were extracted 5 times with 2 mL of hexane and centrifugation (5,000 × g, 3 min, 4°C). The lower phase was acidified with 0.3 mL of HCl 6 N, and the free fatty acids were extracted with 2 mL of hexane by centrifugation (5,000 × g, 3 min, 4°C); to remove residual water, this phase (containing free fatty acids, hexane and residual amounts of water) was filtered through anhydrous sodium sulfate. The solvent was removed using an evaporator (Waterbath B-480 Büchi, Zwitzerland) at 60°C and 500 mmHg vacuum; and the free fatty acids were gravimetrically quantified. Derivatization was performed using a boron trifluoride solution, 0.5 mL (14% in methanol) were added to the fatty acid sample and the mixture was heated in sealed vials and a water bath at 60°C for 20 min, and then rapidly cooled to room temperature. Hexane (3 mL) and a saturated solution of sodium chloride (1 mL) were added, and after centrifugation (5,000 × g, 3 min, 4°C), the upper phase, containing the methyl esters was collected and filtered through anhydrous sodium sulfate to remove residual water. Finally, samples containing the methyl esters and hexane were placed into chromatographic vials. The obtained organic phase was analyzed in a GC-MS system (GC: 6890 N, Agilent Technologies, Wilmington, DE, USA; Mass selective detector: 5973 N, Agilent Technologies, Wilmington, DE, USA). Samples (1 μL) were injected in split mode (30:1) into a fused silica capillary column packed with phenyl methyl siloxane (30 m × 0.250 mm, 0.25 μm, HP-5MS Agilent Technologies, Wilmington, DE, USA), using helium (99.999% purity) as the carrier gas at an on-column flow of 1 mL/min. The temperature of the oven was set at 50°C for 7 minutes, then raised at a rate of 5°C/min to 250°C and held for 15 minutes. The mass detector was operated as follows: the filament was turned on at minute 6, the transfer line from the GC to the MS was maintained at 280°C, the Quadrupole was maintained at 150°C and the ionization source was maintained at 230°C. Electron ionization was operated at 70 eV. The analysis was performed in the full scan mode over the m/z range of 20 to 700.

Initial glucose transport rates were determined according to Utrilla et al. [42]. Cells were harvested from cultures in the mid log phase (14,000 × g for 10 min at 4°C), cooled on ice, washed in 1× BBM salts, and dissolved in the same medium without a carbon source at a cell density of 1 × 10^8^ cells/mL. Because the cells were kept on ice (5 minutes) before the transport experiments, metabolic activity was reduced; nonetheless, it is assumed that the amount of sugar transport protein present is the same as that present during growth under culture conditions. For the [^14^C]-glucose uptake assays, 540 μL of cell suspension were incubated for 15 min at 25°C, and the kinetics experiment was then started by adding 60 μL of [^14^C]-glucose (0.5 mM, 5 mCi/mmol). The suspension was maintained at 25°C with shaking, and 50 μL samples were taken at 0, 5, 10, 20, 40 and 60 min intervals. These samples were then filtered immediately through membrane filters (pore size 0.45 μm) and washed three times with BBM salts. The filters were dried and placed in vials with 5 mL of Ecolite scintillation cocktail (ICN Biomedicals, Costa Mesa, CA, USA). Radioactivity was measured using a scintillation counter (Beckman LS6000IC, Fullerton, CA, USA). The [^14^C]-glucose uptake rates were calculated from the initial linear data based on a plot of intracellular [^14^C]-glucose versus time.

## Abbreviations

BBM: Bold’s basal medium; CCCP: Carbonyl cyanide m-chlorophenyl hydrazine; C/N: Carbon to nitrogen ratio; CO2: Carbon dioxide; DCW: Dry cell weight; QX: Biomass productivity at maximum cell mass produced; QPROT, QLIP, QCARB: Protein, Lipid or carbohydrate productivity at maximum cell mass produced; TAGs: Triacylglycerides; tD: Duplication time; v/v: Volume per volume; w/w: Weight per weight; XMAX: Maximum cell mass produced; YX/GLC: Yield of biomass on glucose; YX/NIT: Yield of biomass on nitrate; μ: Specific growth rate.

## Competing interests

The authors declare that they have no competing interests.

## Authors’ contributions

DM-S carried out the experiments, acquired the data and drafted the manuscript. RT-V performed the fatty acid profile experiments and participated in the results analysis. JK participated in the results interpretation and analysis and critically revised the manuscript. AM conceived the study, participated in the design of the study, performed results interpretation and analysis, and critically revised the manuscript. All authors read and approved the final manuscript.

## Authors’ information

DM-S is currently a PhD student at the Biotechnology Institute-UNAM. She is working with heterotrophic cultures of *Neochloris oleoabundans* to study biomass and lipid production with sugars from lignocellulose hydrolysates. RT-V is an Academic Technician at the Pilot Plant of the Biotechnology Institute-UNAM. He is working with microalgae cultures and laccase production by *Pleurotus ostreatus.* JK is currently Director of Professional Sciences and Assistant Professor at Bellevue University (Nebraska, US). His research background is in the area of developing algae for enhanced biofuel production, as well as photoactive proteins and optogenetics. AM is currently Professor at the Biotechnology Institute-UNAM, his main research interest is in the metabolic engineering and bioprocess development with *Escherichia coli* for biofuels and biopolymer precursors production, as well as physiological studies with oleaginous microalgae for biofuel and feedstock applications.
